# Different facets of alpha and beta diversity of benthic diatoms along stream watercourse in a large near‐natural catchment

**DOI:** 10.1002/ece3.11577

**Published:** 2024-06-13

**Authors:** Naicheng Wu, Guohao Liu, Xinxin Qi, Zongwei Lin, Yixia Wang, Yaochun Wang, Yuying Li, Collins Oduro, Sangar Khan, Shuchan Zhou, Tianjiang Chu

**Affiliations:** ^1^ Department of Geography and Spatial Information Techniques Ningbo University Ningbo China; ^2^ Henan International Joint Laboratory of Watershed Ecological Security in the Water Source Area of the Middle Route of South‐to‐North Water Diversion Project, College of Water Resource and Modern Agriculture Nanyang Normal University Nanyang China; ^3^ Ningbo University Donghai Institute Ningbo University Ningbo China; ^4^ Ningbo University Library (Journal Editorial Department) Ningbo University Ningbo China; ^5^ Hangzhou Academy of Agricultural Sciences Hangzhou China

**Keywords:** common species, functional convergence, nestedness, periphyton, rare species, turnover

## Abstract

Understanding the processes and mechanisms that shape the distribution patterns and variations of biodiversity along spatial gradients continues to be a priority for ecological research. We focused on the biodiversity of benthic diatom communities within a large near‐natural watershed. The objectives are: (1) to explore the overall spatial patterns of benthic diatom biodiversity; (2) to investigate the effects associated with watercourse position and environmental variables, as well as both common and rare species on two facets (i.e., taxonomic and functional) of alpha and beta diversity; and (3) to unveil the mechanisms underlying their spatial variations. Alpha diversity indices along the stream watercourse showed a clear increasing trend from upstream to downstream sites. Results of random forest regression identified conductivity as the primary factor influencing functional alpha diversity, while elevation emerged as the predominant factor for taxonomic alpha diversity. Beta diversity partitioning revealed that taxonomic beta diversity generally exceeded functional beta diversity. These diversity measures exhibited different patterns along the watercourse position: taxonomic beta diversity remained relatively consistent along the watercourse, whereas functional total beta diversity and its two components of middle stream sites were lower than those of upstream and downstream sites. Functional beta diversity was sustained by dominant and common species, while rare species made significant contributions to taxonomic beta diversity. Both taxonomic and functional beta diversity and its components displayed a stronger influence from spatial factors than from local environmental, geo‐climatic, and nutrient variables. Collectively, taxonomic and functional alpha and beta diversity demonstrated distinct responses to the main environmental gradients and spatial factors within our catchment, highlighting their different insights into diatom diversity. Furthermore, research is required to assess the generalizability of our findings to similar ecosystems. In addition, this study presents opportunities for expansion to include other taxa (e.g., macroinvertebrates and fish) to gain a comprehensive understanding of the driving mechanisms behind stream biodiversity.

## Graphical abstract

We explore the effects of watercourse position, common and rare species on taxonomic and functional alpha, and beta diversity in a large near‐natural catchment. In addition, we also reveal the underlying potential mechanisms.

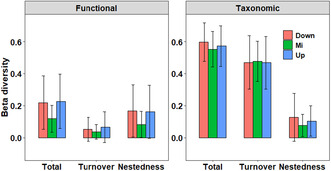

## 1 INTRODUCTION

River ecosystems, home to a myriad of life forms, play a vital role in sustaining life on Earth and offering invaluable products and services. However, the biodiversity within freshwater ecosystems faces a global threat. Since 1970, a staggering 80% decrease in species diversity has been observed in river ecosystems, surpassing the decline in most other ecosystems (Li et al., 2020; Suurkuukka et al., 2014). Human disturbances such as global warming and urbanization have promoted the decline of biodiversity and habitats, the loss of function, and the degradation of river ecosystems (Li et al., 2022). Understanding the processes and mechanisms that shape the distribution patterns and variations of biodiversity along environmental gradients remains a priority for ecological research, especially in the study of community ecology and the potential effects of global change (Aarnio & Soininen, 2021; Jamoneau et al., 2018; Meynard et al., 2011). Meanwhile, clarifying the driving of biodiversity decline is also important for achieving biodiversity conservation goals and developing effective strategies for management decisions (Kang et al., 2018; Zhang et al., 2021; Zhou et al., 2022).

Alpha diversity is the variety and variability of life forms within communities (Grigoropoulou et al., 2022; Laliberte et al., 2020), and species richness is the simplest measure of alpha diversity (Kang et al., 2018). Taxonomic diversity is one of the most studied metrics in community ecological surveys (Zhou et al., 2022). However, co‐occurring species can exhibit different extents of “shared” habitat preference, functional features, or life history (Grigoropoulou et al., 2022; Richter et al., 2021; Wu et al., 2017). Considering species richness or taxonomic diversity could not capture or reflect sufficiently their ecological functions in the ecosystems, functional diversity (the value and the range of variability of functional traits) has been increasingly recommended although they showed some positive correlations with taxonomic diversity (Du et al., 2021; Meynard et al., 2011). It has been found that functional diversity can also contribute to their ecosystem resilience in response to environmental changes (Meynard et al., 2011; Teichert et al., 2018). Functional diversity has been found to provide more measurable and stable characteristics in the responses to environmental changes as well as ecological complementary information (Wu et al., 2019; Zhou et al., 2022). However, alpha diversity is scale‐dependent and their patterns and explanatory factors vary over spatial scales (Bhatta et al., 2018). Therefore, alpha diversity was recommended to be combined with beta diversity to determine community assembly and improve our prediction ability of impacts in environmental changes (Teichert et al., 2018).

Beta diversity, considering the diversity variation across temporal and spatial scales, is a key component of biodiversity. It can provide a powerful tool for understanding the driving forces that underlie the community structure at multiple spatial and temporal scales, as well as for conservation purposes (Baselga & Leprieur, 2015; Villéger et al., 2013; Zhou et al., 2022). It can be partitioned into turnover and nestedness components, and this decomposition can help to detect the mechanisms behind observed beta diversity patterns (Jamoneau et al., 2018; Wu et al., 2023). Turnover involves the replacement of taxa, while nestedness refers to the loss/gain of taxa (Baselga, 2010; Branco et al., 2020; Schmidt et al., 2022). The studies of taxonomic and functional beta diversity can expand our understanding of the underlying mechanisms in community composition variations (Zeng et al., 2022). The comparison of beta diversity in taxonomic and functional facets can help us disentangle the assembly rules of the community (Villeger et al., 2012). For instance, the relationship of beta diversity in different facets and different spatial or environmental scales (even less concerned) can help us understand the maintenance processes of biodiversity (Meynard et al., 2011). Additionally, increasing studies have found that differences in species relative abundance (i.e., common or rare species) or species evenness could also put importance on the stochastic and deterministic processes of beta diversity, which was another important component of diversity (Li et al., 2019; Sebek et al., 2022; Wilsey & Potvin, 2000). Furthermore, effective conservation strategies require comprehensive knowledge considering multiple facets of biodiversity, including alpha/beta components of species richness, taxonomic and functional diversity, and their relationships (Kang et al., 2018).

There are several ecological theories or concepts to explain the spatial patterns of biodiversity, three of which are River Continuum Concept (RCC), landscape filter hypothesis, and metacommunity theory. The RCC is a conceptual framework that describes how physical and ecological characteristics change along the longitudinal gradient of rivers and streams (Vannote et al., 1980). It suggests that stream ecosystems exhibit distinct patterns of structure and functioning from headwaters to larger river systems, which influence the composition and diversity of benthic diatom communities. According to the RCC, local species diversity (i.e., alpha diversity) is expected to be lower in upstream sites compared to larger and more connected downstream sites (Carrara et al., 2012; MacArthur & Wilson, 1963; Schmidt et al., 2022; Vannote et al., 1980). In comparison, beta diversity at upstream sites with heterogeneous and relatively isolated environments receiving fewer migrants should be higher and mainly influenced by turnover components, while at downstream sites where habitats are more interconnected, lower beta diversity should be observed since high dispersal rates give rise to a strong mass effect (Jamoneau et al., 2018; López‐Delgado et al., 2020). The landscape filter hypothesis and metacommunity theory provide a framework for understanding how local communities, such as benthic diatom communities, are connected and influenced by regional processes (Leibold et al., 2004; Poff, 1997). They recognize that the local composition and diversity of diatom communities are influenced by processes operating at multiple scales, from local environment (and bio‐interaction) to regional dynamics that connect different segments. Previous studies have recognized that regional processes can play an important role in shaping local biodiversity of stream organisms (e.g., Lin et al., 2024; Liu et al., 2023; Wu et al., 2021; Wu, Liu et al., 2022; Wu, Lv et al., 2022). Nevertheless, the contributions of spatial versus landscape variables (e.g., local environment, geographic‐climate variables) to various aspects of alpha and beta diversity patterns were still less documented.

In this study, we selected a large Chinese undisturbed catchment to elucidate patterns and potential causes of taxonomic and functional alpha and beta diversity in benthic diatoms along the longitudinal gradient. Particularly, we explored (1) whether or not watercourse position (i.e., up‐, middle, and downstream), common and rare species affected taxonomic and functional alpha and beta diversity patterns of benthic diatoms; (2) evaluated the extent to which environmental and spatial factors (indicating regional processes) impacted taxonomic and functional alpha and beta diversity (and its components). We hypothesize that (H1) alpha diversity increases along the longitudinal gradient from upstream to downstream sites. By contrast, the second hypothesis (H2) is that beta diversity and its turnover component should decrease along the watercourse position (i.e., from up‐ to downstream sites). Furthermore, given that two facets of diversity provided different ecological information (Perez Rocha et al., 2019; Wu et al., 2021; Wu, Wang et al., 2022), for example, functional diversity is less dependent than taxonomic diversity on spatial factors (Soininen et al., 2016), taxonomic and functional alpha and beta diversity (and its components) would respond differently to the main environmental gradients and regional processes in the study area (H3).

## 2 METHODS

### 2.1 Study area

**FIGURE 1:**
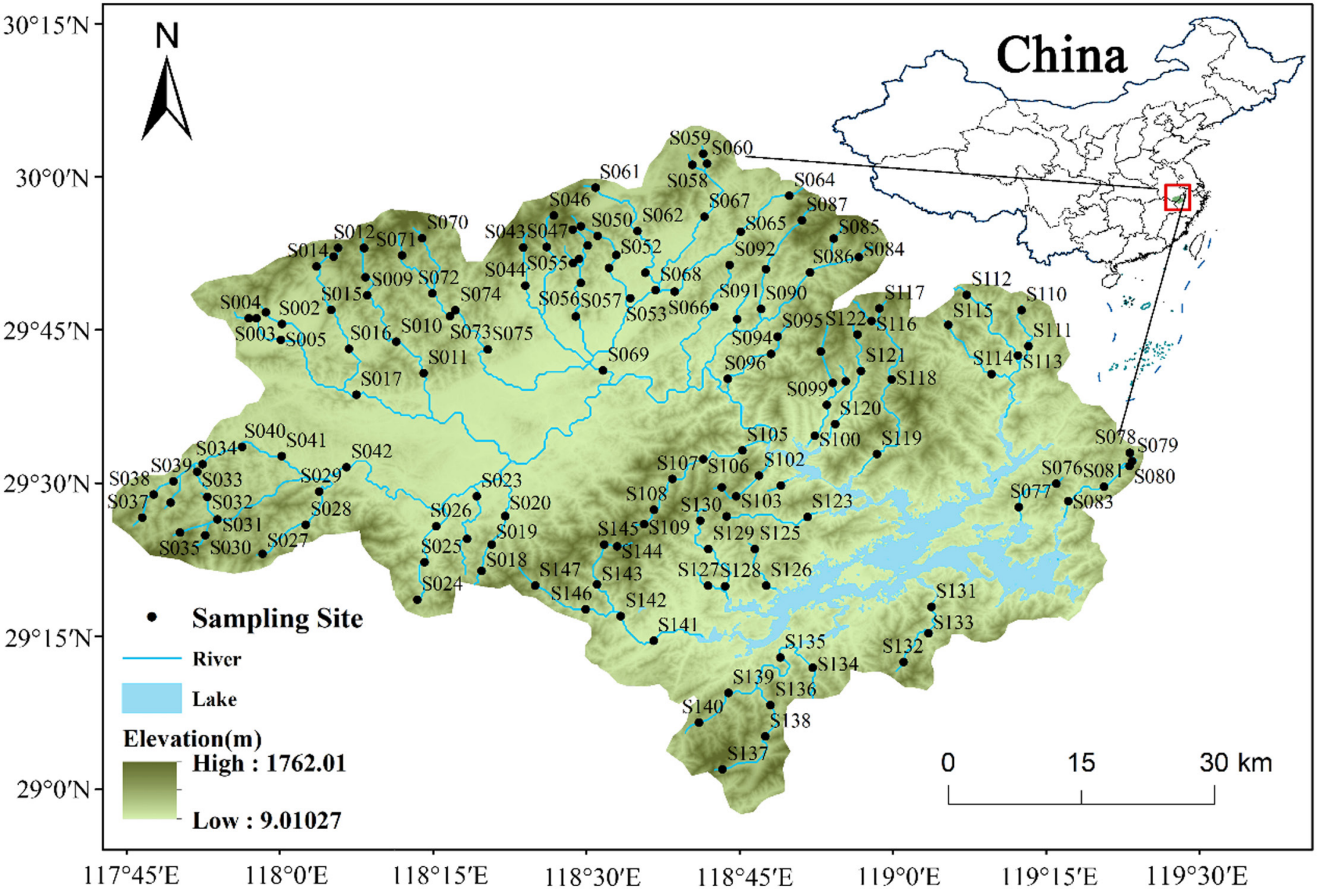
Distribution map of the 147 sampling sites in the Thousand Islands Lake (TIL) catchment, China.

Our study area is situated in the catchment of Thousand Islands Lake (TIL), characterized by a mean annual precipitation of 1430 mm and an expansive catchment area of approximately 10,080 km^2^. Positioned within the subtropical monsoon climate zone in eastern China, our sampling sites span an elevational gradient of over 500 m (ranging from 102 to 648 m), as illustrated in Figure 1. The basin, situated in an ideal location for investigating biodiversity patterns and mechanisms, holds significance for two primary reasons. Firstly, the area boasts a large catchment and exhibits a distinct gradient in climatic and topographic conditions. Secondly, the study area enjoys relative protection from human disturbances, as evidenced by a high mean forest coverage of 96.05% (Table 1). Engaging in research within such gradient patterns proves particularly advantageous for understanding and predicting community responses to natural environmental changes (Lin et al., 2024; Liu et al., 2023; Thrush et al., 2006; Wang et al., 2023; Wu et al., 2021). Catchment lithology is spatially heterogeneous and consists mainly of limestone and sandstone.

**TABLE 1:** Abiotic factors (mean ± SD) used in this study.

Variables	Units	Abbreviation	All sites (*n* = 147)	Down (*n* = 40)	Mi (*n* = 45)	Up (*n* = 62)
*Local environmental variables* (*Loc*)						
Elevation	m	*Elevation*	270.88 ± 131.95	201.9 ± 80.8	219.51 ± 82.91	352.68 ± 143.39
Width	m	*Width*	13.23 ± 14.95	24.14 ± 22.7	12.9 ± 8.79	6.44 ± 4.83
Depth	cm	*Depth*	24.9 ± 11.78	25.33 ± 9.21	27.64 ± 14.02	22.63 ± 11.07
Flow velocity	m/s	*Velocity*	0.41 ± 0.19	0.4 ± 0.18	0.4 ± 0.23	0.43 ± 0.17
pH	–	pH	7.65 ± 0.93	7.45 ± 1	7.88 ± 0.85	7.62 ± 0.9
Water temperature	°C	*WT*	17.23 ± 2.77	17.4 ± 2.65	18.35 ± 2.23	16.32 ± 2.91
Conductivity	μs/cm	*Cond*	167.07 ± 84.77	193.33 ± 95.31	168.49 ± 81.44	149.08 ± 77.47
Habitat score	%	*QHEI*	65.68 ± 9.19	64.97 ± 10.31	63.63 ± 9.4	67.63 ± 7.95
*Geo‐climatic variables* (*Geo*)						
Longitude	°E	X	118.48 ± 0.4	118.4 ± 0.36	118.48 ± 0.39	118.53 ± 0.42
Latitude	°N	Y	29.79 ± 0.23	29.82 ± 0.22	29.71 ± 0.21	29.84 ± 0.24
Annual mean temperature	°C	Bio1	15.39 ± 0.74	15.7 ± 0.51	15.75 ± 0.51	14.93 ± 0.77
Mean diurnal range	°C	Bio2	7.68 ± 0.25	7.73 ± 0.16	7.75 ± 0.24	7.6 ± 0.27
Isothermality	–	*Bio3*	25.04 ± 0.43	25.02 ± 0.32	25.17 ± 0.45	24.96 ± 0.47
Temperature seasonality	°C	Bio4	844.6 ± 12.25	852.13 ± 7.99	846.57 ± 10.72	838.3 ± 12.41
Max temperature of warmest month	°C	Bio5	30.42 ± 1	30.85 ± 0.67	30.86 ± 0.69	29.82 ± 1.06
Min temperature of coldest month	°C	Bio6	−0.25 ± 0.64	−0.04 ± 0.47	0.07 ± 0.5	−0.62 ± 0.65
Temperature annual range	°C	Bio7	30.67 ± 0.52	30.90 ± 0.34	30.79 ± 0.46	30.43 ± 0.57
Mean temperature of wettest quarter	°C	*Bio8*	19.65 ± 0.91	19.78 ± 0.52	19.80 ± 0.52	19.44 ± 1.24
Mean temperature of driest quarter	°C	Bio9	6.69 ± 0.67	6.91 ± 0.5	7.02 ± 0.48	6.3 ± 0.69
Mean temperature of warmest quarter	°C	Bio10	25.46 ± 0.85	25.86 ± 0.55	25.84 ± 0.59	24.93 ± 0.88
Mean temperature of coldest quarter	°C	Bio11	4.69 ± 0.65	4.91 ± 0.48	5.03 ± 0.46	4.31 ± 0.67
Annual precipitation	mm	Bio12	1607.95 ± 83.13	1587.35 ± 69.27	1600 ± 69.75	1627.02 ± 96.06
Precipitation of wettest month	mm	Bio13	275.37 ± 18.49	273.15 ± 16.24	274.31 ± 16.08	277.56 ± 21.24
Precipitation of driest month	mm	Bio14	40.51 ± 1.64	40.25 ± 1.6	40.69 ± 1.57	40.55 ± 1.71
Precipitation seasonality	–	*Bio15*	53.36 ± 1.97	53.78 ± 1.76	53.71 ± 1.89	52.85 ± 2.06
Precipitation of wettest quarter	mm	Bio16	690.99 ± 50.44	687.95 ± 45.61	693.58 ± 46.02	691.06 ± 56.64
Precipitation of driest quarter	mm	Bio17	156.48 ± 6.22	154.78 ± 5.5	156.6 ± 5.68	157.48 ± 6.83
Precipitation of warmest quarter	mm	Bio18	568.34 ± 35.77	557.08 ± 26.35	556.96 ± 26.76	583.87 ± 40.83
Precipitation of coldest quarter	mm	Bio19	192.35 ± 10.34	190.98 ± 9.75	194.82 ± 9.74	191.44 ± 10.97
Slope of the sampling site	°	*Slope*	11.01 ± 8.38	7.38 ± 5.62	9.93 ± 8.85	14.12 ± 8.44
Aspect of the sampling site	°	*Aspect*	200.44 ± 108.44	206.62 ± 109.19	178.02 ± 109.69	212.73 ± 105.8
Forest	%	*Forest*	96.05 ± 6.57	93.38 ± 8.35	95.14 ± 7.2	98.43 ± 3.14
Cropland	%	Cropland	3.51 ± 6.01	5.83 ± 7.58	4.34 ± 6.72	1.4 ± 2.84
Shrubs	%	Shrubs	0.01 ± 0.03	0.01 ± 0.01	0.01 ± 0.03	0.02 ± 0.04
Grassland	%	Grassland	0.02 ± 0.05	0.02 ± 0.02	0.03 ± 0.05	0.02 ± 0.05
Open water%	%	Water	0.06 ± 0.24	0.09 ± 0.22	0.09 ± 0.36	0.01 ± 0.08
Impervious	%	Impervious	0.35 ± 0.66	0.67 ± 0.97	0.38 ± 0.45	0.12 ± 0.41
*Nutrient variables* (*Nut*)						
Chemical oxygen demand	mg/L	*CODMn*	1.12 ± 0.36	1.25 ± 0.42	1.09 ± 0.32	1.05 ± 0.32
Ammonia–nitrogen	mg/L	*NH3‐N*	0.11 ± 0.04	0.13 ± 0.05	0.1 ± 0.02	0.11 ± 0.05
Nitrate–nitrogen	mg/L	*NO3‐N*	1.02 ± 0.55	1.04 ± 0.5	0.98 ± 0.49	1.03 ± 0.63
Phosphate	mg/L	*PO4‐P*	0.01 ± 0.01	0.02 ± 0.02	0.01 ± 0.01	0.01 ± 0.01
Total nitrogen	mg/L	*TN*	1.80 ± 0.60	1.85 ± 0.56	1.81 ± 0.57	1.76 ± 0.65
Total phosphorus	mg/L	*TP*	0.05 ± 0.05	0.07 ± 0.04	0.06 ± 0.07	0.04 ± 0.02
TN/TP ratio	–	NPR	45.02 ± 23.38	34.28 ± 19.27	45.57 ± 26.29	51.55 ± 21.18
*Spatial factors* (*Spa*)						
MEM1	–	*MEM1*	0 ± 1	0.08 ± 0.96	−0.3 ± 0.78	0.16 ± 1.13
MEM2	–	*MEM2*	0 ± 1	−0.14 ± 1.04	0.13 ± 1.06	0 ± 0.94
MEM3	–	*MEM3*	0 ± 1	−0.01 ± 0.77	0.03 ± 1.17	−0.01 ± 1.03
MEM4	–	*MEM4*	0 ± 1	−0.08 ± 1.19	−0.01 ± 0.97	0.06 ± 0.9
MEM5	–	*MEM5*	0 ± 1	−0.22 ± 0.82	0.1 ± 1.18	0.07 ± 0.98
…	–	*…*				
MEM39	–	*MEM39*	0 ± 1	−0.04 ± 1.17	0.04 ± 0.87	−0.01 ± 0.99

*Note*: Variables in *italic* were finally selected after removing variables with strong intercorrelation (i.e., Spearman's rank |*r*| >0.8).

### 2.2 Data collection

#### 2.2.1 Local environmental and nutrient variables

A comprehensive sampling effort was undertaken in March and April 2021, encompassing a total of 147 sites distributed across the study area (Figure 1). Geographical coordinates and elevation data were recorded at each site using a Trimble‐Juno SB GPS receiver. Simultaneously, a suite of local environmental variables was measured *in situ* to provide a holistic understanding of the ecological context. Water temperature (WT), water depth (Depth), flow velocity (Velocity) using the Global Water Flow Probe FP201, pH, conductivity (Cond) utilizing the YSI Multiparameter instrument professional plus, and stream width (Width) were all quantified. In addition, the Qualitative Habitat Evaluation Index (QHEI) was computed, incorporating 10 distinct categories. These categories encompassed in‐stream habitat complexity, substrate composition, bank stability, the range of combined water depth and velocity, channel sinuosity, visual inspection of water cleanliness, water quantity, biodiversity of riparian plants, land‐use types, and the environmental stress originating from human activities (Taft & Koncelik, 2006). Each of the 10 QHEI categories underwent a visual assessment and received a score within the range of 0–10, where lower scores denoted lower habitat quality, and higher scores indicated superior habitat quality. The final QHEI score, a cumulative representation of these categories, serves as an indicator of overall habitat quality, with higher scores reflecting more favorable conditions. The QHEI has been widely used in assessing habitat conditions of previous studies including this catchment (e.g., Liu et al., 2023, 2024), which were thus employed in this investigation. Concurrently, stream surface water samples were systematically collected and acid‐fixed for subsequent laboratory analysis. In the controlled laboratory environment, a suite of nutrient variables, including total phosphorus (TP), total nitrogen (TN), chemical oxygen demand (CODMn), ammonia nitrogen (NH3‐N), nitrate–nitrogen (NO3‐N), and soluble reactive phosphorus (PO4‐P), were analyzed. TN/TP (NPR) was the ratio between TN and TP. To streamline our analysis and highlight the importance of nutrients, we grouped the seven nutrient variables (Nut) separately from the remaining environmental variables, designated as local environmental (Loc) (Table 1).

#### 2.2.2 Collection and processing of benthic diatoms

At each site, about nine representative stones were selected, and benthic algae in each stone were carefully brushed and rinsed with distilled water and then collected in containers. The collected samples were preserved with 99.7% alcohol. In the laboratory, permanent diatom slides were made by hydrogen peroxide (30% H_2_O_2_ solution) method (Wu et al., 2017) and a minimum of 300 valves were counted for each slide. Following the identifications of Hu et al. (1980) and Zhu and Chen (2000), benthic diatoms were identified to the lowest possible taxonomic level (mostly to species level). To investigate the relationships between species traits and the surrounding environment and calculate functional alpha and beta diversity, we collected data on three widely used diatom species trait categories (i.e., cell size, guild, and life forms) resulting in 15 traits using 0–1 for presence and absence (for details see Table S1 and previous studies: Passy, 2007; Rimet & Bouchez, 2012; Wu et al., 2017).

#### 2.2.3 Geographic‐climatic (Geo) variables

Climatic, land use, and topographic data were consolidated into a unified category termed geographic‐climatic (hereafter as geo‐climatic) variables. Land‐use data were obtained from Yang and Huang (2021), providing land cover information for China with a spatial resolution of 30 m. Adapting to the study area's characteristics, land‐use types were reclassified into six categories: forest, cropland, shrubs, grassland, water, and impervious. Topographic variables, including slope and aspect, were extracted from a prior study (Amatulli et al., 2018). Aspect captures information on the north–south and east–west orientation of each sampling site, whereas slope indicates the steepness of the stream along a longitudinal gradient (Amatulli et al., 2018). In addition, 19 bio‐climatic variables (Bio1 to Bio19) were obtained from the WorldClim 2 database (Fick & Hijmans, 2017), including annual mean temperature, maximum temperature in the warmest month, annual precipitation of each site (a full list of 19 variables is shown in Table 1).

#### 2.2.4 Spatial factors (Spa)

Distance‐based Moran's Eigenvector Maps (MEMs), in which smaller eigenvalues indicate broad‐scale spatial patterns, whereas larger eigenvalues indicate fine‐scale patterns, was widely used to investigate the contribution of regional processes to community structuring (e.g., Declerck et al., 2011; Li et al., 2021; Liu et al., 2022). Here, *dbmem* function in R package *adespatial* was applied to calculate spatial factors (MEMs) and 39 MEMs were obtained (Dray et al., 2020).

### 2.3 Data analysis

We conducted analyses at two levels. At the catchment level, we used the whole data set (i.e., 147 sites). Simultaneously, at a finer resolution, the site was partitioned into three groups along the longitudinal gradient, namely, upstream, middle, and downstream. Sampling sites were categorized based on Strahler stream order, which is commonly used in hydrology, geomorphology, and river management to study the characteristics and dynamics of river networks (Strahler, 1957). It provides a way to quantify and analyze the size and complexity of stream networks. Higher stream orders typically represent larger streams with more tributaries, while lower orders represent smaller, headwater streams. Those falling in the first stream order (62 sites) were grouped as upstream, while sites in the second stream order comprised the middle category (45 sites). The remaining sites (in third and fourth stream orders) were designated as downstream (40 sites). This stratification enables the testing of hypotheses H1 and H2, exploring whether diversity patterns differ based on watercourse position. In addition, we split species based on their relative abundances into dominant, common, and rare species in order to explore their different contributions to biodiversity variation. Rare species contributed less than 1% to the entire community, common species contributed more than 1% (Tornés et al., 2021), and dominant species had a contribution exceeding 5% (Ma et al., 2023).

We first calculated taxonomic species richness and Pielou evenness (i.e., TRic and TEve), two informative indices to represent local taxonomic diversity, using *diversity* function in *vegan* package (Oksanen et al., 2019) of R (version 4.2.2) (R Core Team, 2022). Then, we calculated functional richness and evenness (i.e., FRic and FEve) based on species composition and trait matrix using *dbFD* function in R package *FD* (Laliberté & Legendre, 2010). FRic measures the volume of the functional space occupied by the community, while FEve represents the regularity of the distribution of species abundances and dissimilarities in functional space (Villéger et al., 2008). Both are increasingly considered as important functional diversity indices that shed light on the mechanistic link between community and ecosystem functioning.

Taxonomic and functional beta diversities were calculated based on the presence–absence matrix, utilizing the Sørensen dissimilarity index and illustrated in flowcharts (see Figures S1 and S2). The decomposition of taxonomic beta diversity into turnover and nestedness components was achieved using the *beta*.*pair* function from the R package *betapart* (Baselga & Orme, 2012). Concurrently, the computation of functional turnover and nestedness components followed the methodology proposed by Perez Rocha et al. (2019) and Wu et al. (2021), employing the *functional*.*beta*.*pair* function in the R package *betapart*. Subsequently, taxonomic and functional beta diversities, along with their respective components, were computed for up‐, middle, and downstream sites using the same methodology. Furthermore, exploration included the decomposition of common and rare species, employing the identical approach, to unravel their relative contributions to beta diversity.

To test our first hypothesis (H1) that whether alpha diversity increases from up‐ to downstream sites, we ran the non‐parametric Kruskal–Wallis tests among the three groups. For testing hypothesis H2 and discerning potential differences in taxonomic and functional beta diversity components among watercourse positions, permutational analysis of multivariate dispersions (PERMDISP) was employed, utilizing the *betadisper* function in the R *vegan* package. In instances of significance, subsequent pairwise Tukey's honestly significant difference (HSD) tests were executed to identify significant differences between watercourse positions, specifically up‐, middle, and downstream sites.

To test hypothesis H3 and examine the key environmental and spatial factors determining spatial patterns of taxonomic and functional biodiversity, we employed random forest regression (RFR) (Genuer et al., 2010) and distance‐based redundancy analysis (db‐RDA) (Legendre & Anderson, 1999) for alpha diversity indices and beta diversity matrices, respectively. RFR is a popular non‐parametric machine‐learning algorithm and has been widely employed in recent ecological studies due to its flexibility and robustness over traditional linear regression models (Genuer et al., 2010; Li et al., 2023; Qu et al., 2022). RFR generates a number of decision trees (here, 1000 trees) and identifies the most important predictor variables for target alpha diversity index. Mean squared error (MSE) and *R*‐squared (*R* ^2^) were used to evaluate the RFR model performance. RFR was run using the *randomForest* function in the R package *randomForest* (Breiman, 2001), followed by *importance* function from the same package to rank the variable importance. The method with db‐RDA can be based on any dissimilarity or distance matrices, although it is similar to redundancy analysis (Legendre & Legendre, 2012). Hence, it has been used by several similar studies (e.g., Branco et al., 2020; Ding et al., 2021; López‐Delgado et al., 2020; Perez Rocha et al., 2018; Pozzobom et al., 2021; Wu et al., 2021), which were comparable with ours. Following the step‐by‐step procedures (see also the work flowchart in Figures S1 and S2), we removed the variables with strong intercorrelation (i.e., Spearman's rank |*r*| >0.8) to reduce multicollinearity and then performed a global test (by ANOVA at a significance level of α = 0.05) for each facet of beta diversity by using *capscale* function in the vegan package; last, a forward selection (by *forward*.*sel* function in R package *adespatial*) when the global test was significant was conducted to obtain a final set of four abiotic factors (i.e., Loc, Geo, Nut, and Spa). Variation partitioning analysis (VPA, in R package *vegan*) (Borcard et al., 1992) was carried out to quantify the relative roles of different abiotic factors to each facet of beta diversity and its components. Results from VPA were shown by Venn diagrams, which is a standard way to show the fractions explained purely by each dataset as well as by their intersections (e.g., López‐Delgado et al., 2020; Perez Rocha et al., 2018; Wu et al., 2021).

## 3 RESULTS

Abiotic factors varied considerably within the catchment (Table 1). For instance, elevation ranged from 102 to 648 m (average: 271 m), WT ranged from 7.87 to 23.27°C (average: 17.23°C), pH ranged from 5.51 to 9.50 (average: 7.65), river width ranged from 1.23 to 104.77 m (average: 13.23 m), river depths ranged from 9 to 80 cm (average: 25 cm), and flow velocity ranged from 0.01 to 1.07 m/s (average: 0.41 m/s). NH3‐N ranged from 0.03 to 0.40 mg/L (average: 0.11 mg/L), NO3‐N ranged from 0.21 to 3.40 mg/L (average: 1.02 mg/L), and PO4‐P ranged from 0.00 to 0.06 mg/L (average: 0.01 mg/L). TP ranged from 0.01 to 0.51 mg/L (average: 0.05 mg/L), and TN ranged from 0.81 to 4.20 mg/L (average: 1.80 mg/L). From up‐ to downstream sites, elevation, river width, Cond, percentage of cropland, CODMn, TN, and TP increased while percentage of forest and slope decreased (Table 1).

Overall, we observed 119, 127, and 128 diatom species in the up‐, middle, and downstream sites, respectively, and a total of 168 species in the whole basin with an average number of 20 species per site (range: 5–49), which belonged to 55 genera and 29 families. The most dominant species, *Achnanthidium delmontii*, was present at 135 out of 147 sites, with a mean relative abundance of 21.35%. Its relative abundance exhibited a decreasing trend along the watercourse position, with the highest mean relative abundance observed in upstream sites (24.18%), followed by middle (22.00%) and downstream sites (15.82%). Additionally, *Achnanthidium eutrophilum*, *Cocconeis placentula*, *Gomphonema pumilum* v*ar*. *rigidum*, and *Melosira varians* were identified as the dominant species, each having a relative abundance exceeding 5%. *M*. *varians* showed the same decreasing trend as *A*. *delmontii* from up‐ to downstream sites. In contrast, relative abundances of *C*. *placentula* and *G*. *pumilum var*. *Rigidum* showed an opposite trend (i.e., increasing from up‐ to downstream sites), whereas *A*. *eutrophilum* showed higher relative abundances in the up‐ and middle streams (6.05% and 6.07%, respectively) and lower in the downstream sites (5.20%). Except for the dominant species, there were still 17 diatom species with relative abundance >1%: *Achnanthidium latecephalum*, *Cymbella affinis var*. *procera*, *Encyonema minutum*, *Gomphonema elegantissimum*, and *Navicula subalpina* increased along the watercourse position, respectively; *Fistulifera saprophila*, *Fragilaria vaucheriae*, *Amphora inariensis*, and *Gomphonema olivaceum var*. *calcareum* reached highest relative abundances in the upstream sites, while *Gomphosphenia holmquistii*, *Achnanthidium catenatum*, *Gomphonema lagenula*, *Cymbella lancettula*, *Sellaphora bacillum*, and *Ulnaria danica* peaked in the middle sites.

### 3.1 Alpha diversity pattern in relation to abiotic factors

**FIGURE 2:**
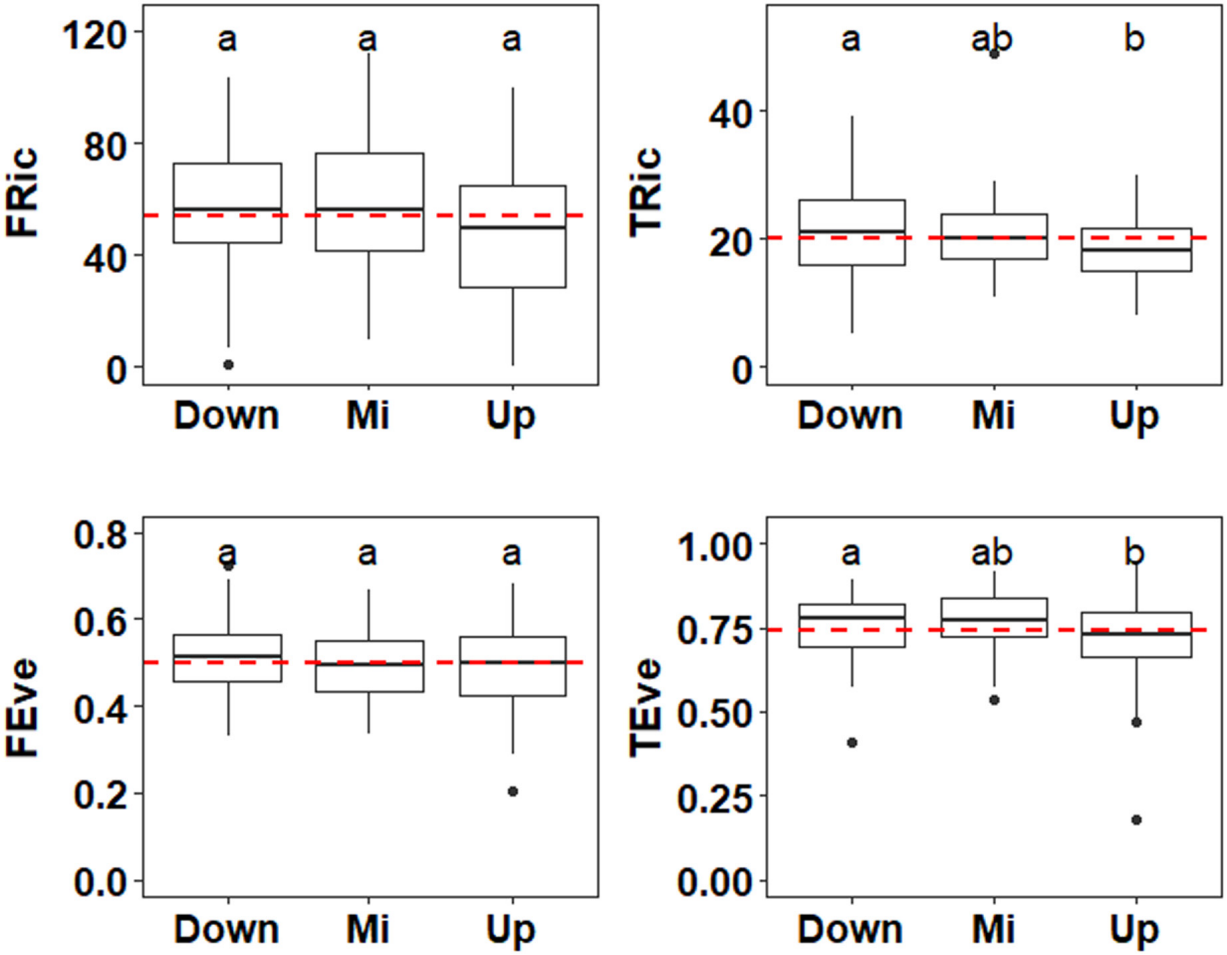
Results of alpha diversity values for the downstream (Down), middle (Mi), and upstream (Up) sites, respectively. Boxes show interquartile ranges (25th and 75th percentiles) and medians (middle lines); whiskers are non‐outlier ranges beyond boxes, and dots are outliers. Horizontal red dashed lines represent average values. Differences among groups were assessed using Kruskal–Wallis tests; different letters above boxes indicate significant differences (*p* < .05). FEve, functional evenness; FRich, functional richness; TEve, taxonomic evenness; TRich, taxonomic species richness.

The alpha diversity indices exhibited a distinct increasing trend from upstream to downstream sites, as depicted in Figure 2. This observation confirms a longitudinal gradient of escalating alpha diversity along the watercourse. Both taxonomic alpha diversities (TRic and TEve) were significantly lower at upstream sites compared to downstream sites. Notably, no significant difference was observed in functional alpha diversities (FRic and FEve) among the upstream, middle, and downstream sites (Figure 2). Furthermore, the relationships among various alpha diversity indices were relatively low, except for TRic vs. FRic (see Figure S3), emphasizing the different ecological information offered by these indices.

**FIGURE 3:**
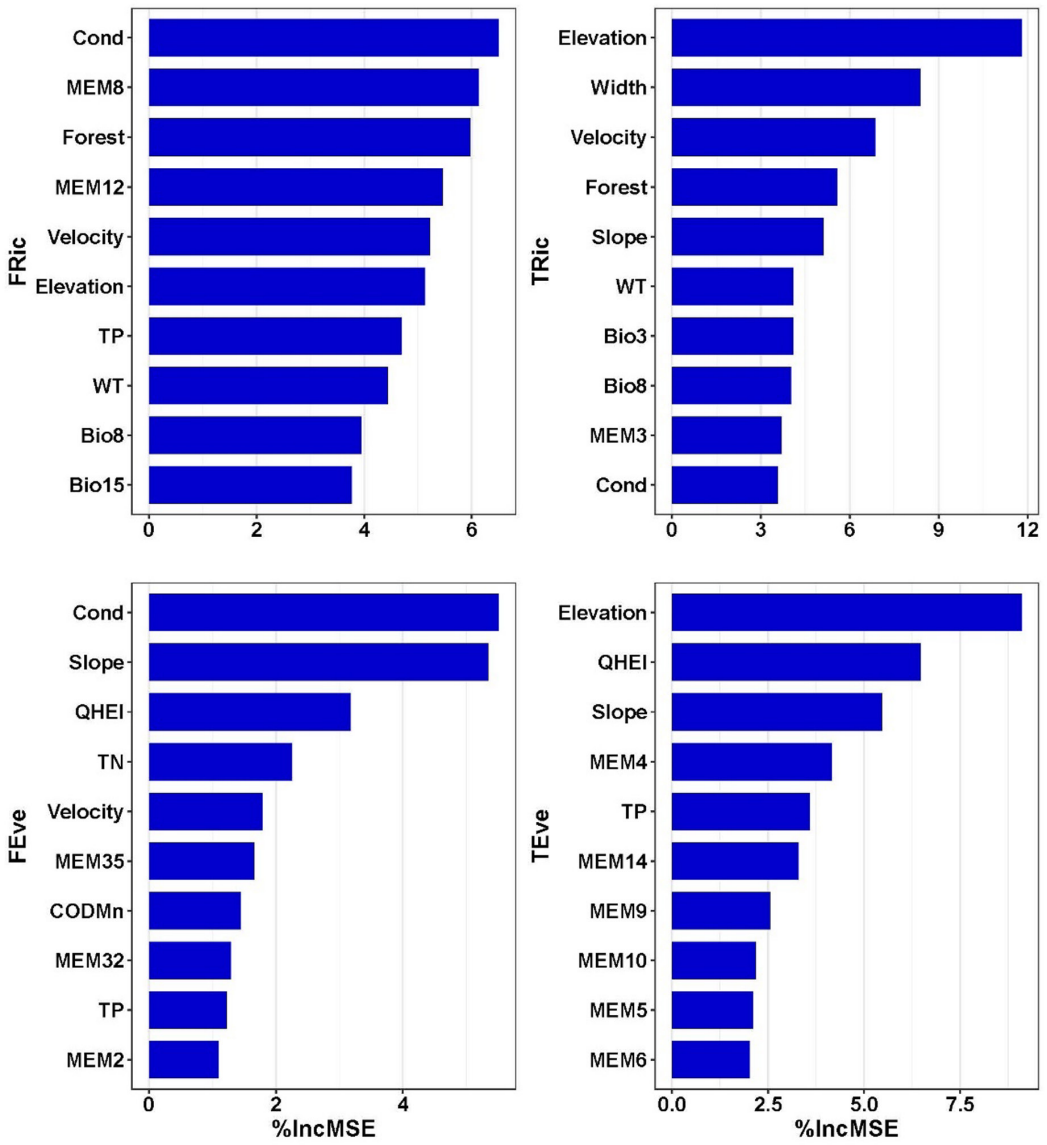
Results of random forest regressions showing the 10 most important predictors for functional and taxonomic alpha diversity indices. %IncMSE, increased in mean squared error (%); FEve, functional evenness; FRich, functional richness; TEve, taxonomic evenness; Trich, taxonomic species richness.

The random forest regression (RFR) demonstrated good model fits, as indicated by MSE (5.26, 0.002, 93.2, 0.002) and R^2^ (0.95, 0.96, 0.96, 0.96) for TRic, TEve, FRic, and FEve, respectively. Results from RFR highlighted conductivity as the primary factor influencing functional alpha diversity (FRic and FEve), with fine‐scale spatial factors (e.g., MEM12, MEM32, and MEM35) and local habitat quality (e.g., velocity, TP) following closely (see Figure 3). In contrast, elevation was a predominate factor for TRic and TEve; except for elevation, local habitat quality (such as width, velocity, slope), geo‐climatic, and spatial factors (e.g., MEM3, MEM4, MEM14) were also important for the variation of taxonomic alpha diversity. Taxonomic and functional alpha diversity shared several important co‐predictors. For instance, elevation, conductivity, forest, velocity, WT, and Bio8 (mean temperature of the wettest quarter) were co‐factors of the alpha richness (i.e., TRic and FRic), while slope, QHEI, and TP were the co‐factors of alpha evenness (i.e., TEve and FEve).

### 3.2 Beta diversity pattern and its main drivers

**FIGURE 4:**
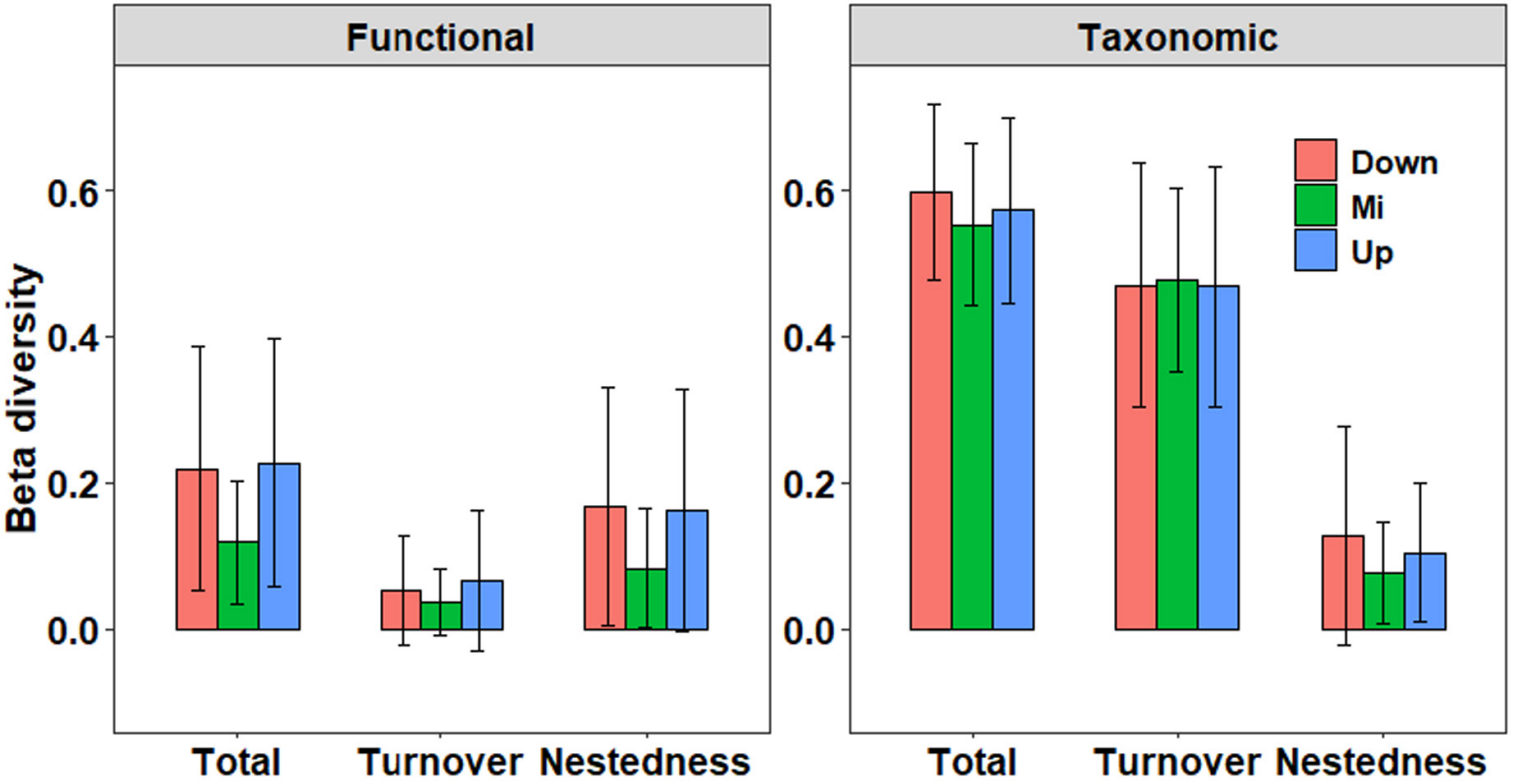
Statistic description (Mean ± SD) of taxonomic and functional beta diversity components (i.e., total, turnover, and nestedness) for the downstream (Down), middle (Mi), and upstream (Up) sites, respectively.

**FIGURE 5:**
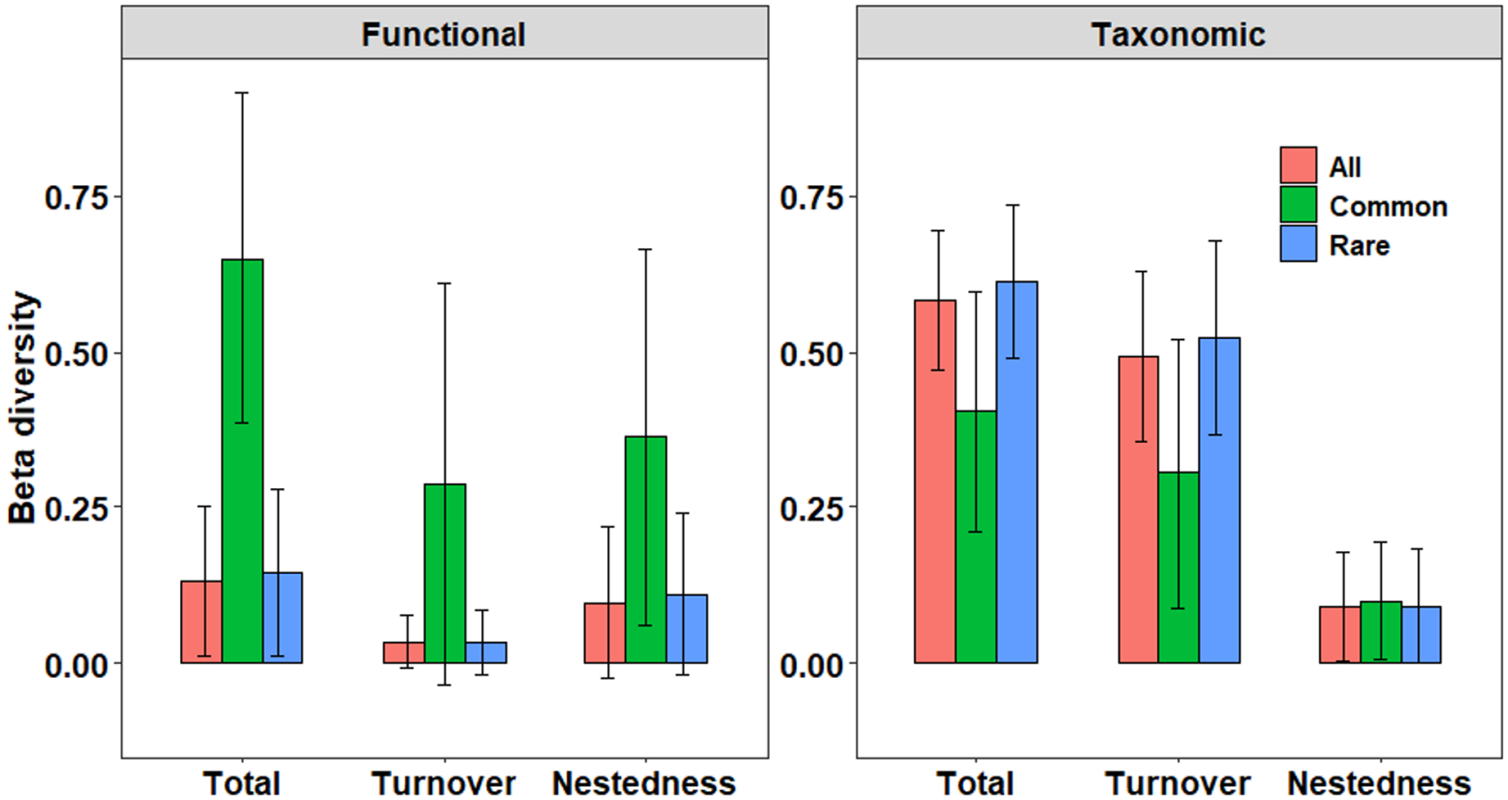
Statistic description (Mean ± SD) of taxonomic and functional beta diversity components (i.e., total, turnover, and nestedness) for all, common, and rare species, respectively.

Beta diversity partitioning revealed that taxonomic beta diversity was generally higher than functional beta diversity, both of which showed different patterns along the watercourse position. Taxonomic beta diversity did not differ significantly along the watercourse. In contrast, functional total beta diversity and its two components of middle stream sites were lower than those of upstream and downstream sites (Figure 4), although statistical differences were only detected between upstream–downstream and middle–downstream sites of the turnover component (Table S2). This means some different diatom species may share the “same” functional traits in the TIL catchment, while functional convergence was more obvious in the middle stream sites. Taxonomic beta diversity was mainly contributed by turnover (81.76%, 86.32%, and 78.72% for up‐, middle, and downstream sites, respectively), whereas functional beta diversity was mostly related to nestedness (71.39%, 69.86%, and 75.97% for up‐, middle, and downstream sites, respectively) (Figure 4). Common species occupying a wide range of habitats explained most of the variation of functional beta diversity. In contrast, rare species enhanced the taxonomic alpha diversity but contributed less to the functional diversity (Figure 5). Moreover, turnover and nestedness of common species contributed almost equivalently to its functional total beta diversity. In comparison, functional total beta diversity of rare species was controlled mostly by nestedness (Figure 5).

The db‐RDA analyses revealed that both environmental and spatial factors played a crucial role in shaping both facets of beta diversity and their components in this catchment, as detailed in Tables S3 and S4. However, it is noteworthy that the significant variables differed between facets and beta diversity components. For instance, model selections on Loc highlighted that water temperature (WT), conductivity (Cond), and elevation were important predictors for taxonomic total beta diversity, while elevation and WT were identified as significant predictors for functional total beta diversity. Model selections on Nut indicated TN, CODMn, and PO4‐P significantly explained variation in taxonomic total beta diversity, while no variables were picked up for functional total beta diversity. Similarly, model selections on Geo and Spatial resulted in different significant variables for both facets of beta diversity and its components. VPA results showed that the pure contribution of Spatial factors (1–6%) was higher than those of Loc (1–2%), Geo (0–1%), and Nut (0) to taxonomic beta diversity and its components; for functional beta diversity and its components, the pure contribution of Spatial (1–4%) was also larger than Loc (0–2%) and Geo (0–1%). In summary, as expected by H3, taxonomic and functional alpha and beta diversity (and its components) responded differently to the main environmental gradients and spatial factors in our catchment. However, the explained variations remained very low. For functional beta diversity, the models explained only 5% of total, 3% of turnover, and 7% of nestedness, respectively. For taxonomic beta diversity, the significant global models explained 11% of the total, 14% of turnover, and 11% of nestedness, respectively.

## 4 DISCUSSION

### 4.1 Taxonomic and functional alpha diversity of benthic diatoms

In this study, taxonomic richness showed a similar trend with taxonomic evenness. Previous studies widely reported richness coupled with evenness, and this was because evenness is normally dependent on relative abundances (Balseiro & Waisfeld, 2014; Gosselin, 2006; Tornés et al., 2021). High values of both taxonomic and functional evenness revealed that the diatom community in our study was relatively even (Figure 2). Larger species evenness reflects a greater range of adaptive strategies that are more equally involved in producing biomass (Cerabolini et al., 2010). A similar finding was reported by the studies of Aarnio and Soininen (2021) and González‐Paz et al. (2022). In our study, the relative even distribution can also be detected by the structure of diatom species composition. The diatom community was dominated by five widespread species (with relative abundance >5%: in order of importance, *A*. *delmontii*, *G*. *pumilum var*. *Rigidum*, *M*. *varians*, *C*. *placentula*, and *A*. *eutrophilum*). Dominant species due to “mass ratio” effect contributed most to the biomass and had a greater influence on ecosystem processes. In this study, although the five species dominated the community, their relative abundances were low (*A*. *delmontii* with 21.35% and the others with ~5%). Communities in which dominant species with lower relative abundance have higher community evenness; conversely, communities with increasing relative abundance of dominant species can decrease the community evenness (Cerabolini et al., 2010; Wilsey & Potvin, 2000); additionally, rare species in our study are accounting for a high number of alpha richness (~87% of total richness), which induced diatom community exhibited relatively even.

Diatoms, due to their rapid growth rate and tiny size, are especially sensitive to environmental variations and, with different functional traits, could adapt with different strategies (Perez Rocha et al., 2019). In our study, significantly increasing trends of diatom taxonomic richness and evenness from up‐ to downstream sites were detected, confirming H1. Strong heterogeneity along the watercourse position was also detected in other studies, such as the diatom study in a Chinese urban stream (Chen et al., 2016), the fish fauna in the Mekong River, China (Zhang et al., 2021), and riverine benthic invertebrate assemblages of central Germany (Tonkin et al., 2015). Environmental and spatial factors such as Cond, elevation, Width, Forest, and WT were important predictors for taxonomic and functional alpha diversity indices (Figure 3). Conductivity was found to be one of the most influential variables for diatom species (Benito et al., 2018; González‐Paz et al., 2022), probably because it links to variations in lithology. Elevation was also found to be a significant factor in the diatom community in the Xiangxi River, China (Wu et al., 2014), and tropical South American freshwaters (Benito et al., 2018).

### 4.2 Taxonomic and functional beta diversity of benthic diatoms

Surprisingly, our results found that both taxonomic and functional total beta diversity and its two components did not show a progressive decreasing from upstream to downstream sites (Figure 4), challenging H2 and previous findings (e.g., Finn et al., 2011; Jamoneau et al., 2018; Schmidt et al., 2022). Hence, H2 was not supported. The conventional pattern of higher beta diversity in upstream sites compared to downstream sites, referred to as the “classical pattern,” was not universally applicable in freshwater ecosystems. Recent studies investigating taxonomic beta diversity (Aspin & House, 2022; Tonkin et al., 2015) have documented that this classical pattern could be changed or even reversed as the case of this study. A study with riverine benthic invertebrate assemblages of central Germany found a trend of decreasing beta diversity from headwaters to large rivers, but there was no significant difference between the three sections (Tonkin et al., 2015). One potential reason is the well‐protected habitats of our whole catchment, as indicated by the large forest coverage and low nutrient contents (Table 1), which provide isolated micro‐habitats in middle and downstream sites supporting even higher niche dimensionality than upstream sites. This could be verified by the fact that most of our sampling sites (107 out of 147 sites) are located in stream orders 1 and 2. Furthermore, as diatom beta diversity was negatively related to nutrient enrichment (Jamoneau et al., 2018; Leboucher et al., 2019), the low and homogeneous nutrient concentrations among different watercourse positions may result in similar taxonomic beta diversity. Besides, the widespread low‐head dams at the middle and downstream sites might be another potential reason to increase their beta diversity by creating more heterogeneous patches. These results emphasize the importance of further research on beta diversity patterns in well‐preserved catchments.

Furthermore, the study revealed that both the total and turnover of functional beta diversity were significantly lower than their taxonomic counterparts, consistent with findings from several previous studies (e.g., Perez Rocha et al., 2019, Wu et al., 2021, Wu, Wang et al., 2022). This pattern of functional convergence has been observed across various taxa, including butterfly (Zhou et al., 2022), birds (Zeng et al., 2022), macroinvertebrates (Wu, Liu et al., 2022), fish (Wu, Lv et al., 2022), and cerambycid beetles (Bezerra‐Gusmao et al., 2022). Similar to other beta diversity studies of the diatom community, functional nestedness was a bit higher than turnover of functional beta diversity (Perez Rocha et al., 2019; Wang et al., 2022; Wu et al., 2021). This suggests a prevalent influence of stochastic processes, such as extinction and recolonization, in shaping the functional dynamics of diatom communities (Perez Rocha et al., 2019).

The result that taxonomic turnover contributed principally to the total taxonomic beta diversity has been widely reported in different taxa such as stream benthic algae (Jamoneau et al., 2018; Wu et al., 2021), herbivorous insects (Kuchenbecker et al., 2021), and bird community (Zeng et al., 2022). A high turnover signifies historical fingerprints in more stable areas, such as geographical barrier formation and environmental sorting (deterministic processes), while high nestedness implies prevalent events of extinction and recolonization, such as dispersal limitation and environmental filtering (stochastic processes) (Dapporto et al., 2014). In this study, the alpha diversity of the diatom community may be controlled by geographical barriers (e.g., waterfalls, dams) and environmental sorting. Additionally, the high proportion of rare species of diatom community in our study contributed a lot to the variation of taxonomic beta diversity. Rare species rather than common species could contribute the most to local and regional alpha and gamma diversity (Draper et al., 2019). In this study, the taxonomic total and turnover of rare species were higher than those of common species (Figure 5). This was consistent with the studies of Li et al. (2019) and Haack et al. (2022). In the study of Li et al. (2019), rare taxa of soil bacteria exhibited a higher species turnover than common taxa because common bacteria taxa occupy wider niche breadths and are strongly influenced by deterministic filtering (higher turnover of beta diversity), while rare taxa were primarily controlled by stochastic processes (higher nestedness of beta diversity). Rare Beetle species were found to have a higher taxonomic beta diversity than common ones, and in which turnover was also always dominant over richness differences (Haack et al., 2022). However, in the study of Draper et al. (2019), dominant tree species common only in a single forest type maintained the regional patterns of beta diversity and played a key role in structuring western Amazonian tree communities, and Richardson et al. (2012) found dominant vascular plant species in alpine ecosystem of New Zealand had a narrow range of similar trait values and led to trait convergence, in which functional turnover (2.9%) were extremely lower than taxonomic turnover (75%).

An interesting finding was that turnover and nestedness of common species contributed almost equivalently to its functional total beta diversity, while the functional total beta diversity of rare species was driven mostly by nestedness (Figure 5). This can be explained by the difference in the habitat range of the community composition (Draper et al., 2019; Richardson et al., 2012). Abundant taxa were found to maintain a larger number of functions than rare taxa (Zhang et al., 2022). Hence, it was expected that the common species of diatom assemblages in our study predominated the variation of functional beta diversity (Figure 5). Another interesting finding was that the roles of common and rare species in taxonomic and functional beta diversity were different: The contribution of rare species to functional beta diversity was low, while their contribution to taxonomic beta diversity was high (Figure 5). Common and rare species had different dispersal abilities and habitat specializations. Hence, they obey distinct assembly rules. For example, rare species may be more spatially restricted and have higher habitat specialization, so are much more vulnerable to environmental changes than common species (Benone et al., 2022). Furthermore, previous studies have found that dominant, common, and rare species can contribute different weights to the variation of beta diversity (Sebek et al., 2022) and altering species relative abundances and/or changes to the size of species pool can also influence local diversity variation (Blowes et al., 2022). Hence, these findings highlight the need for further research on the relative roles of common and rare species to biodiversity patterns in order to provide deeper insights into underlying mechanisms.

### 4.3 Diversity of benthic diatoms in relation to abiotic factors

**FIGURE 6:**
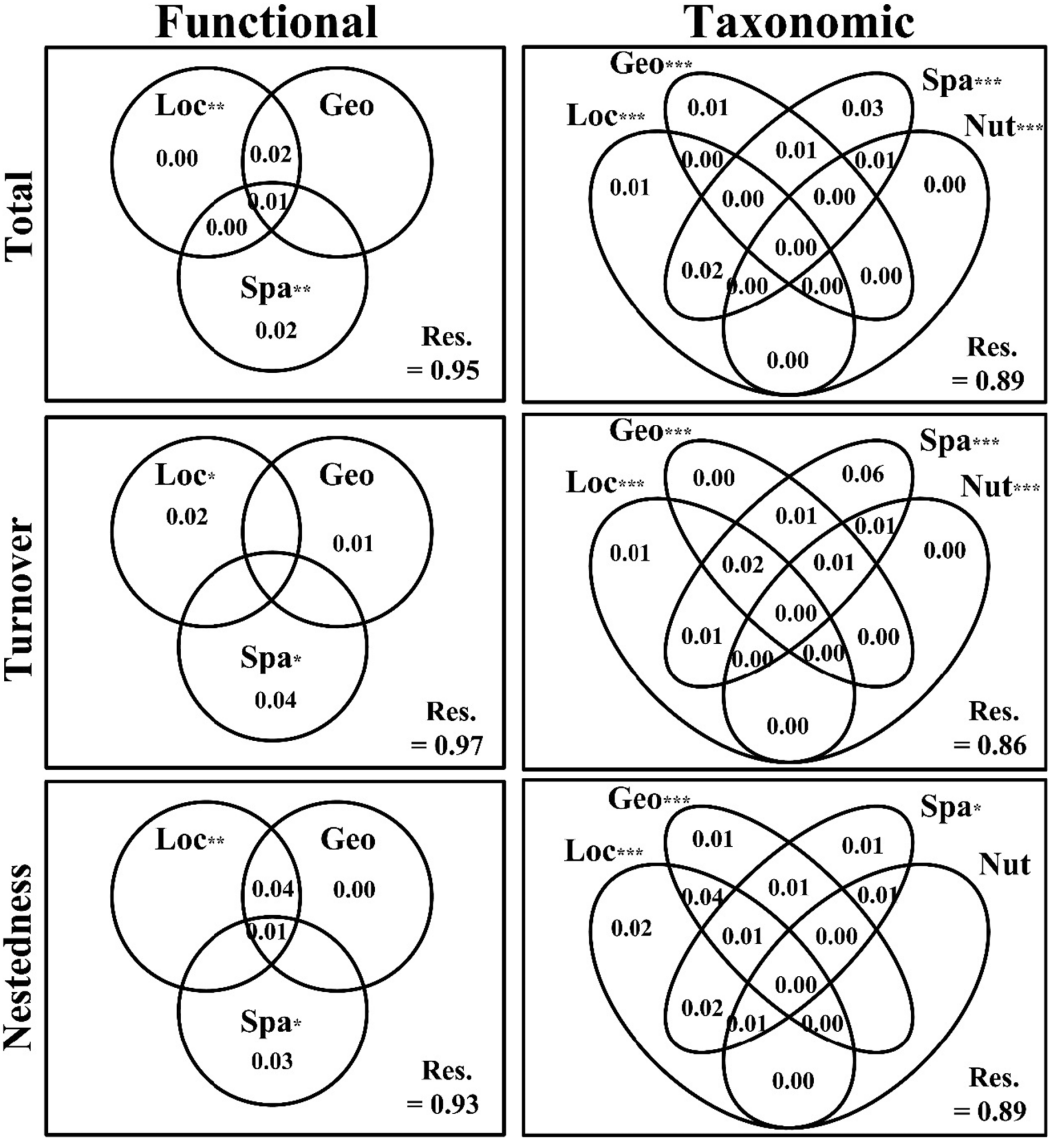
Variance partitioning analysis (VPA) of functional and taxonomic beta diversity components (i.e., total, turnover, nestedness) by local environmental (Loc), geo‐climatic (Geo), nutrient (Nut), and spatial (Spa) variables. Adjusted *R* ^2^ is presented (values <0 are not shown). Nut was removed from the VPA of functional beta diversity components since no variables were selected. ***p < .001; **p < .01, *p < .05.

The results from db‐RDA and VPA align with the expectations set by H3, indicating that taxonomic and functional alpha and beta diversity, along with their components, exhibit distinct responses to the primary environmental gradients and spatial factors within our catchment. This outcome is consistent with the established understanding of the complementarity between taxonomic and functional facets, emphasizing the importance of considering multiple dimensions of biodiversity in both fundamental and applied research (Heino & Tolonen, 2017; Li et al., 2023; Wang et al., 2023; Wu, Wang et al., 2022). The VPA results (depicted in Figure 6) revealed that spatial factors exerted a more significant influence on both taxonomic and functional beta diversity, as well as their components, compared to local environmental, geo‐climatic, and nutrient variables. This finding aligns with several recent studies (Jamoneau et al., 2018; Leboucher et al., 2020; Lin et al., 2024; Liu et al., 2023; Wu et al., 2021), which found a crucial role of spatial factors in determining stream benthic organism patterns across large spatial scales.

It must be noted that our abiotic factors explained very few variations of both taxonomic and functional beta diversity components with residuals of 86–97% (Figure 6), although these values were comparable to those of previous studies (e.g., Branco et al., 2020; López‐Delgado et al., 2020; Perez Rocha et al., 2018). This implied that other variables not considered here, such as environmental regime changes (Wu et al., 2023) and biotic interactions (predation, grazing, facilitation and/or competition), might account for a big fraction of beta diversity's variation. Several recent studies have proved the importance of environmental regimes in structuring the biodiversity pattern of river algal communities (e.g., Guo et al., 2020; Wu et al., 2023; Wu, Wang et al., 2022). Guo et al. (2020) calculated short‐period flow regime indices (2 and ~4 weeks before the sampling date) and found that these indices overrode physicochemical factors in structuring riverine diatom composition. Regarding bio‐interaction, a study of diatom–bacteria interactions has confirmed that the presence of bacteria changed the composition and decreased the productivity of benthic diatoms (Koedooder et al., 2019). Another recent study has highlighted the effect of biotic interactions, which was often overlooked in previous research, on stream macroinvertebrates (Liu et al., 2023). Therefore, future investigations that include the aforementioned variables may advance our understanding of biodiversity–environment relationships.

## 5 CONCLUSION

Along the watercourse position in Thousand Islands Lake (TIL) catchment, the diatom community displayed an increasing alpha diversity from up‐ to downstream sites. However, taxonomic and functional beta diversity showed inconsistent patterns in the study area, demonstrating their different information for biodiversity conservation and policymakers. In addition, common and rare species acted differently to taxonomic and functional beta diversity. Therefore, more studies are needed to explore whether our findings are generalizable to other similar ecosystems. Spatial factors played a major role in structuring taxonomic and functional beta diversity, suggesting that strategies for biodiversity management should focus on promoting fluvial connectivity. In addition, a relatively lower explanation power of VPA in this study implied that some unmeasured variables, such as environmental regime changes and biotic interactions, may play important roles in explaining variation in beta diversity. Our findings call for a broader contextualization of adaptive strategies across taxa and highlight the need to extend the scope of investigations to include additional taxonomic groups such as zooplankton, macroinvertebrates, and stream fish. Our study propels us toward a more nuanced and comprehensive understanding of the intricate web of interactions shaping stream biodiversity and function. This knowledge is indispensable for effective conservation strategies and the sustainable management of freshwater ecosystems worldwide.

## AUTHOR CONTRIBUTIONS

**Naicheng Wu:** Conceptualization (equal); data curation (equal); funding acquisition (equal); investigation (equal); resources (equal); writing – original draft (equal). **Guohao Liu:** Formal analysis (supporting); investigation (equal); writing – review and editing (supporting). **Xinxin Qi:** Investigation (supporting); methodology (supporting); writing – review and editing (supporting). **Zongwei Lin:** Data curation (supporting); investigation (supporting); writing – review and editing (supporting). **Yixia Wang:** Investigation (equal); methodology (equal); visualization (equal); writing – review and editing (supporting). **Yaochun Wang:** Investigation (equal); methodology (equal); writing – review and editing (equal). **Yuying Li:** Formal analysis (supporting); methodology (supporting); writing – review and editing (supporting). **Collins Oduro:** Methodology (equal); writing – review and editing (equal). **Sangar Khan:** Funding acquisition (equal); writing – review and editing (equal). **Shuchan Zhou:** Conceptualization (equal); methodology (equal); supervision (equal); visualization (equal); writing – review and editing (equal). **Tianjiang Chu:** Methodology (equal); project administration (equal); validation (equal); writing – review and editing (equal).

## CONFLICT OF INTEREST STATEMENT

The authors declare no conflict of interest.

## Supporting information


Appendix S1:

## Data Availability

All data are uploaded as supporting material.
