# Supplementary material for: Different facets of alpha and beta diversity of benthic diatoms along stream watercourse in a large near‐natural catchment
Source: Ecol Evol. 2024 Jun 13;14(6):e11577. doi: 10.1002/ece3.11577 (PMC11169757; doi:10.1002/ece3.11577)
Supplement: Supplementary file 1 — Appendix S1: [file ECE3-14-e11577-s001.zip › Supporting information-R02_Wu-plain text.docx]

Supporting information

Different facets of alpha and beta diversity of benthic diatoms along stream watercourse in a large near-natural catchment

**Fig. S1:** A flow-chart of taxonomic beta diversity analyses.


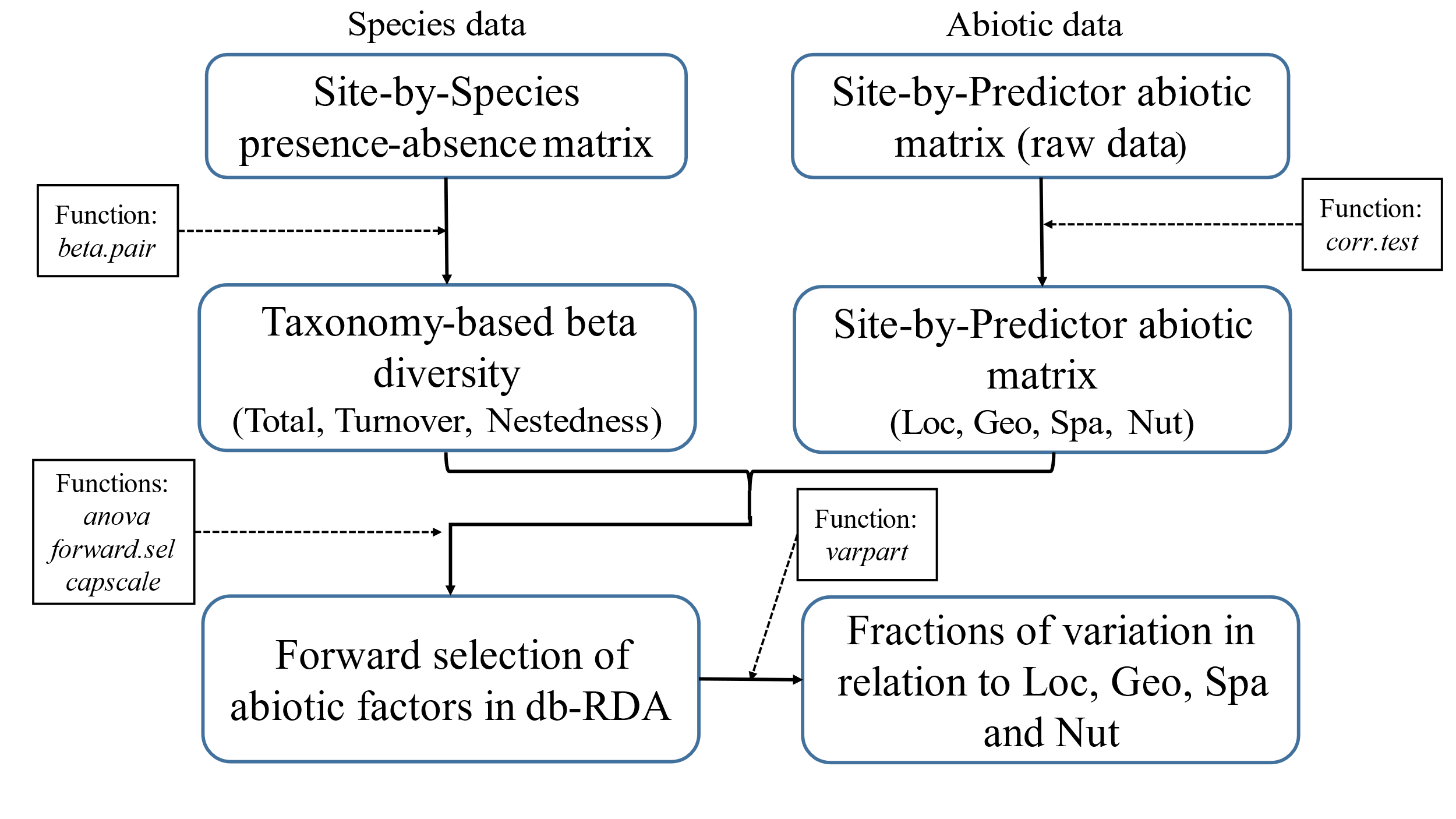


**Fig. S2:** A flow-chart of functional beta diversity analyses.


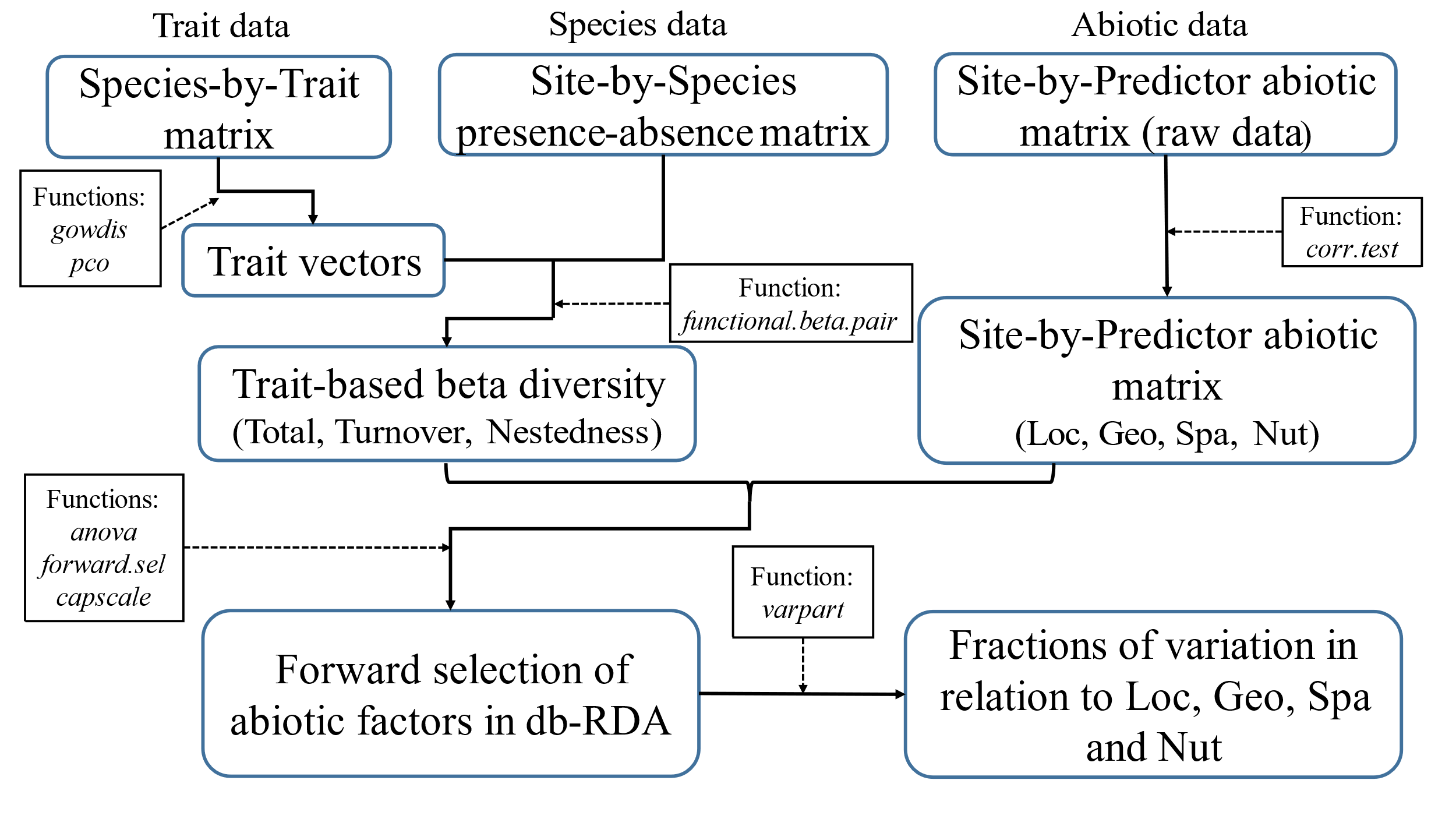


**Fig. S3**: Relationships among different alpha diversity indices.


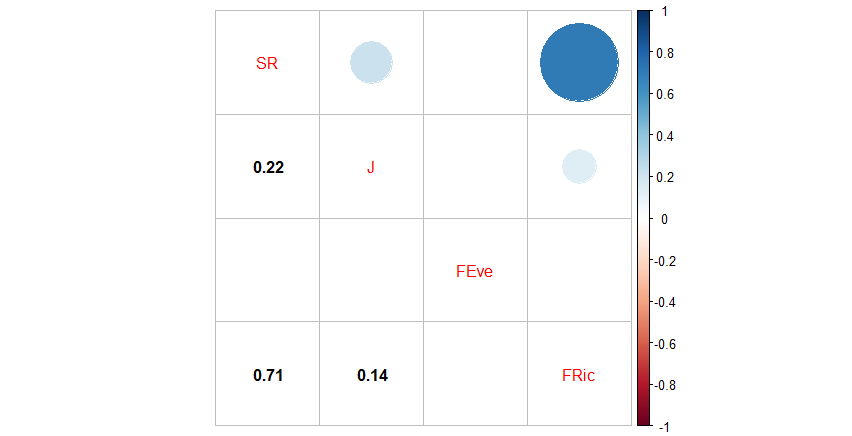


SR: taxonomic richness TRic; J: taxonomic evenness, Teve; FEve: functional evenness; FRic: functional richness.

**Table S1:** Diatom traits, their categories and codes used in this study.

| **Traits** | **Categories** | **Codes** |
| --- | --- | --- |
| 1. Cell size (Berthon et al., 2011; Rimet and Bouchez, 2012; Wu et al., 2017) | Nano (0– 100 µm^3^) | CellSize01 |
|  | Micro (100– 300 µm^3^) | CellSize02 |
|  | Meso (300– 600 µm^3^) | CellSize03 |
|  | Macro (600– 1,500 µm^3^) | CellSize04 |
|  | Large (≥1,500 µm^3^) | CellSize05 |
| 2. Guild (Passy, 2007; Rimet and Bouchez, 2012) | Low profile taxa | LowPro |
|  | High profile taxa | HigPro |
|  | Motile taxa | MotTax |
|  | Planktonic taxa | PlaTax |
| 3. Life form (Biggs et al., 1998; Lange et al., 2016; Wagenhoff et al., 2013; Witteveen et al., 2020) | Unicellular life form | LF_Uni |
|  | Filamentous life form | LF_Fil |
|  | Colonial life form | LF_Col |
|  | Low attachment | ATT_Low |
|  | Medium attachment | ATT_Med |
|  | High attachment | ATT_Hig |

**Table S2:** Differences in beta diversity and its components (i.e., total, turnover, nestedness) among different watercourse positions (i.e., Up: upstream; Mi: middle; Down: downstream). PERMDISP and TurkeyHSD Post-Hoc were used. Diff stands for difference between river sections. Statistically significant values (p<0.05) are shown in bold font.

| **Functional** | | | **Taxonomic** | | |  |
| --- | --- | --- | --- | --- | --- | --- |
|  | diff | p-adj |  | diff | p-adj | |
| **Total** |  |  | **Total** |  |  | |
| Mi-Up | -0.003 | 0.992 | Mi-Down | -0.016 | 0.625 | |
| Down-Up | 0.033 | 0.218 | Up-Down | -0.005 | 0.939 | |
| Down-Mi | 0.035 | 0.152 | Up-Mi | 0.010 | 0.778 | |
| **Turnover** | |  | **Turnover** | |  |  |
| Mi-Up | -0.001 | 0.948 | Mi-Down | 0.011 | 0.876 | |
| Down-Up | 0.011 | **0.026** | Up-Down | 0.004 | 0.982 | |
| Down-Mi | 0.012 | **0.007** | Up-Mi | -0.007 | 0.933 | |
| **Nestedness** | |  | **Nestedness** | |  |  |
| Mi-Up | -0.003 | 0.989 | Mi-Down | -0.026 | 0.131 | |
| Down-Up | 0.022 | 0.482 | Up-Down | 0.013 | 0.545 | |
| Down-Mi | 0.025 | 0.367 | Up-Mi | 0.013 | 0.538 | |

**Table S3:** Results of forward selection of local environmental (Local), geo-climatic (Geo), nutrient (Nut) and spatial (Spatial) variables for taxonomic β-diversity components, respectively. The selected variables are in the order in which they were selected in the forward selection procedure. AdjR^2^Cum (cumulative adjusted R-square), F and p values are shown. Significance is expressed as * p < 0.05, ** p < 0.01, *** p < 0.001.

| Total | | | | Turnover | | | | Nestedness | | | |
| --- | --- | --- | --- | --- | --- | --- | --- | --- | --- | --- | --- |
| Variables | AdjR^2^Cum | F | p | Variables | AdjR^2^Cum | F | p | Variables | AdjR^2^Cum | F | p |
| Local*** | | | | Local*** | | | | Local*** | | | |
| WT | 0.056 | 9.581 | 0.001 | WT | 0.053 | 9.180 | 0.001 | Elevation | 0.082 | 14.118 | 0.001 |
| Cond | 0.076 | 4.180 | 0.001 | Cond | 0.070 | 3.608 | 0.007 |  |  |  |  |
| Elevation | 0.091 | 3.458 | 0.005 | Elevation | 0.076 | 2.036 | 0.046 |  |  |  |  |
| Geo*** | | | | Geo*** | | | | Geo*** | | | |
| Bio15 | 0.043 | 7.530 | 0.001 | Bio15 | 0.042 | 7.343 | 0.001 | Slope | 0.048 | 8.366 | 0.001 |
| Bio3 | 0.066 | 4.643 | 0.002 | Bio3 | 0.059 | 3.632 | 0.005 |  |  |  |  |
| Forest | 0.073 | 2.037 | 0.038 |  |  |  |  |  |  |  |  |
| Spatial*** | | | | Spatial*** | | | | Spatial* | | | |
| MEM2 | 0.057 | 9.826 | 0.001 | MEM2 | 0.055 | 9.436 | 0.001 | MEM36 | 0.020 | 4.033 | 0.020 |
| MEM1 | 0.086 | 5.664 | 0.001 | MEM1 | 0.092 | 7.000 | 0.001 | MEM12 | 0.039 | 3.747 | 0.010 |
| MEM4 | 0.109 | 4.667 | 0.001 | MEM7 | 0.116 | 4.850 | 0.001 | MEM16 | 0.056 | 3.678 | 0.017 |
| MEM7 | 0.131 | 4.564 | 0.001 | MEM4 | 0.138 | 4.739 | 0.001 | MEM24 | 0.069 | 3.019 | 0.047 |
| MEM8 | 0.148 | 3.909 | 0.001 | MEM8 | 0.154 | 3.611 | 0.002 | MEM1 | 0.082 | 3.045 | 0.032 |
| MEM15 | 0.160 | 2.924 | 0.009 | MEM3 | 0.168 | 3.398 | 0.004 | MEM30 | 0.095 | 3.017 | 0.028 |
| MEM14 | 0.168 | 2.397 | 0.019 | MEM33 | 0.176 | 2.420 | 0.018 | MEM6 | 0.108 | 2.918 | 0.046 |
| MEM36 | 0.175 | 2.240 | 0.038 | MEM16 | 0.185 | 2.429 | 0.020 | MEM33 | 0.119 | 2.806 | 0.039 |
| MEM9 | 0.182 | 2.179 | 0.047 | MEM9 | 0.193 | 2.350 | 0.030 | MEM3 | 0.130 | 2.771 | 0.047 |
| MEM20 | 0.188 | 1.998 | 0.041 | MEM31 | 0.200 | 2.200 | 0.039 |  |  |  |  |
| MEM16 | 0.194 | 1.998 | 0.044 | MEM10 | 0.205 | 1.988 | 0.036 |  |  |  |  |
|  |  |  |  | MEM13 | 0.211 | 1.942 | 0.045 |  |  |  |  |
|  |  |  |  | MEM14 | 0.217 | 1.955 | 0.042 |  |  |  |  |
| Nut*** | | | | Nut*** | | | | Nut | | | |
| TN | 0.019 | 3.812 | 0.003 | CODMn | 0.015 | 3.164 | 0.004 | TN | 0.012 | 2.809 | 0.050 |
| CODMn | 0.041 | 4.367 | 0.002 | TN | 0.032 | 3.631 | 0.006 |  |  |  |  |
| PO4.P | 0.059 | 3.735 | 0.006 |  |  |  |  |  |  |  |  |

Bio3= Isothermality, Bio15= Precipitation Seasonality.

**Table S4:** Results of forward selection of local environmental (Local), geo-climatic (Geo), nutrient (Nut) and spatial (Spatial) variables for functional β-diversity components, respectively. The selected variables are in the order in which they were selected in the forward selection procedure. AdjR^2^Cum (cumulative adjusted R-square), F and p values are shown. Significance is expressed as * p < 0.05, ** p < 0.01, *** p < 0.001.

| Total | | | | Turnover | | | | Nestedness | | | |
| --- | --- | --- | --- | --- | --- | --- | --- | --- | --- | --- | --- |
| Variables | AdjR^2^Cum | F | p | Variables | AdjR^2^Cum | F | p | Variables | AdjR^2^Cum | F | p |
| Local** | | | | Local* | | | | Local** | | | |
| Elevation | 0.072 | 12.412 | 0.002 | Cond | 0.041 | 7.166 | 0.001 | Elevation | 0.059 | 10.075 | 0.002 |
| WT | 0.089 | 3.556 | 0.036 | Elevation | 0.056 | 3.420 | 0.007 |  |  |  |  |
|  |  |  |  | Depth | 0.066 | 2.452 | 0.041 |  |  |  |  |
| Geo | | | | Geo | | | | Geo | | | |
| Bio5 | 0.068 | 11.636 | 0.001 | Forest | 0.015 | 3.278 | 0.014 | Bio5 | 0.054 | 9.407 | 0.001 |
| Spatial** | | | | Spatial* | | | | Spatial* | | | |
| MEM24 | 0.043 | 7.494 | 0.013 | MEM12 | 0.018 | 3.716 | 0.010 | MEM24 | 0.041 | 7.258 | 0.013 |
| MEM8 | 0.064 | 4.282 | 0.034 | MEM1 | 0.034 | 3.399 | 0.011 |  |  |  |  |
|  |  |  |  | MEM24 | 0.050 | 3.416 | 0.015 |  |  |  |  |
|  |  |  |  | MEM15 | 0.066 | 3.359 | 0.011 |  |  |  |  |
|  |  |  |  | MEM8 | 0.075 | 2.483 | 0.033 |  |  |  |  |
|  |  |  |  | MEM16 | 0.084 | 2.386 | 0.042 |  |  |  |  |

Bio5= Max Temperature of Warmest Month (°C).

References:

Berthon V, Bouchez A, Rimet F. Using diatom life-forms and ecological guilds to assess organic pollution and trophic level in rivers: a case study of rivers in south-eastern France. Hydrobiologia 2011; 673: 259-271.

Biggs BJF, Stevenson RJ, Lowe RL. A habitat matrix conceptual model for stream periphyton. Archiv für Hydrobiologie 1998; 143: 21-56.

Lange K, Townsend CR, Matthaei CD. A trait-based framework for stream algal communities. Ecology and evolution 2016; 6: 23-36.

Passy SI. Diatom ecological guilds display distinct and predictable behavior along nutrient and disturbance gradients in running waters. Aquatic Botany 2007; 86: 171-178.

Rimet F, Bouchez A. Life-forms, cell-sizes and ecological guilds of diatoms in European rivers. Knowledge and Management of Aquatic Ecosystems 2012; 406: 01-14.

Wagenhoff A, Lange K, Townsend CR, Matthaei CD. Patterns of benthic algae and cyanobacteria along twin-stressor gradients of nutrients and fine sediment: a stream mesocosm experiment. Freshwater Biology 2013; 58: 1849-1863.

Witteveen NH, Freixa A, Sabater S. Local and regional environmental factors drive the spatial distribution of phototrophic biofilm assemblages in Mediterranean streams. Hydrobiologia 2020; 847: 2321-2336.

Wu N, Dong X, Liu Y, Wang C, Baattrup-Pedersen A, Riis T. Using river microalgae as indicators for freshwater biomonitoring: Review of published research and future directions. Ecological Indicators 2017; 81: 124-131.
